# Genome Sequence of a Urease-Positive *Campylobacter lari* Strain

**DOI:** 10.1128/genomeA.01191-15

**Published:** 2015-10-15

**Authors:** Richard J. Meinersmann, James L. Bono, Rebecca L. Lindsey, Linda L. Genzlinger, Vladimir N. Loparev, Brian B. Oakley

**Affiliations:** a USDA Agricultural Research Service, Athens, Georgia, USA; b USDA Agricultural Research Service, Clay Center, Nebraska, USA; c Centers for Disease Control and Prevention, Atlanta, Georgia, USA; d College of Veterinary Medicine, Western University of Health Sciences, Pomona, California, USA

## Abstract

*Campylobacter lari* is frequently isolated from shore birds and can cause illness in humans. Here, we report the draft whole-genome sequence of a urease-positive strain of *C. lari* that was isolated in estuarial water on the coast of Delaware, USA.

## GENOME ANNOUNCEMENT

*Campylobacter lari* is a member of the thermophilic cluster of the genus *Campylobacter*, and as such is closely related to *C. jejuni*, *C. coli*, *C. upsaliensis*, *C. insulaenigrae*, and *C. helviticus*. A genomic sequence has previously been described for *C. lari* RM2100 (GenBank number NC012039), an isolate of the nalidixic acid-resistant thermophilic campylobacters (NARTC) group that was originally isolated from a toddler with watery diarrhea (1). *C. lari* frequently has been isolated from shore birds and survives better than other *Campylobacter* species in surface waters (2).

Using a previously described procedure for isolating *Campylobacter* from environmental water (3), we isolated a *Campylobacter*-like organism in estuarine waters near Slaughter Beach, Delaware, USA (N38.91398°, W75.30843°). For selection, this isolation included incubation at 42°C with cefoperazone. *C. lari* is not usually cefoperazone-resistant, and initial PCR assays to identify the isolate were positive for a *Campylobacter* genus-specific probe and negative for a specific probe for *C. lari* glyA (4). However, sequencing of the 16S ribosomal fragment gene revealed a sequence characteristic of *C. lari*.

The genomic sequence of the strain was determined by sequencing with Pacific Biosciences XL-C2 chemistries and assembled with long-versus-long error correction (PacBioToCA), followed by the Celera Assembler version 7 into a single circular contig of 1,563,032 bases. This sequence was used as a scaffold for assembling data from three runs on a Roche 454 Jr. using the GS FLX Rapid Library prep kit followed by the GS Junior Titanium emPCR (Lib-L) kit (Roche, Branford, CT, USA) (257,885 total fragments, 404-base average read length) and there were less than one base per thousand disagreements. The sequence was also confirmed by genomic optical mapping (OpGen) with approximately 97% agreement. There was no evidence of the presence of any plasmids.

The Slaughter Beach strain sequence had a GC ratio of 29.8%. Annotation was performed by Glimmer (5), which predicted 1,584 open reading frames (ORFs). Three ribosomal operons were identified, each with tRNA-Ile and tRNA-Ala genes in the 16S–23S intergenic fragment.

The sequence showed almost exact synteny with the sequence for *C. lari* RM2100 with several insertions and deletions. A larger fragment present in the Slaughter Beach strain carried two ORFs with significant similarity to urease genes. Thus, the Slaughter Beach strain is a member of the urease-producing thermophilic campylobacters (UPTC) group. The *glyA* gene of the Slaughter Beach strain differed from that of *C. lari* RM2100 by 3.7%, critically differing at the GC-clamps of the 3ʹ end of both primers used for species identification.

### Nucleotide sequence accession number.

This whole-genome shotgun project has been deposited in DDBJ/ENA/GenBank under the accession number CP011372. The version described in this paper is the first version.
